# Host Specificity of Ovine *Bordetella parapertussis* and the Role of Complement

**DOI:** 10.1371/journal.pone.0130964

**Published:** 2015-07-09

**Authors:** Sara E. Hester, Laura L. Goodfield, Jihye Park, Heather A. Feaga, Yury V. Ivanov, Liron Bendor, Dawn L. Taylor, Eric T. Harvill

**Affiliations:** 1 Department of Veterinary and Biomedical Sciences, The Pennsylvania State University, University Park, Pennsylvania, United States of America; 2 Graduate Program in Biochemistry, Microbiology and Molecular Biology, The Pennsylvania State University, University Park, Pennsylvania, United States of America; 3 Graduate Program in Immunology and Infectious Disease, The Pennsylvania State University, University Park, Pennsylvania, United States of America; 4 Graduate Program in Bioinformatics and Genomics, The Pennsylvania State University, University Park, Pennsylvania, United States of America; 5 Graduate Program in Genetics, The Pennsylvania State University, University Park, Pennsylvania, United States of America; University Medical Center Utrecht, NETHERLANDS

## Abstract

The classical bordetellae are comprised of three subspecies that differ from broad to very limited host specificity. Although several lineages appear to have specialized to particular host species, most retain the ability to colonize and grow in mice, providing a powerful common experimental model to study their differences. One of the subspecies, *Bordetella parapertussis*, is composed of two distinct clades that have specialized to different hosts: one to humans (*Bpp_hu_*), and the other to sheep (*Bpp_ov_*). While *Bpp_hu_* and the other classical bordetellae can efficiently colonize mice, *Bpp_ov_* strains are severely defective in their ability to colonize the murine respiratory tract. *Bpp_ov_* genomic analysis did not reveal the loss of adherence genes, but substantial mutations and deletions of multiple genes involved in the production of O-antigen, which is required to prevent complement deposition on *B*. *bronchiseptica* and *Bpp_hu_* strains. *Bpp_ov_* lacks O-antigen and, like O-antigen mutants of other bordetellae, is highly sensitive to murine complement-mediated killing *in vitro*. Based on these results, we hypothesized that *Bpp_ov_* failed to colonize mice because of its sensitivity to murine complement. Consistent with this, the *Bpp_ov_* defect in the colonization of wild type mice was not observed in mice lacking the central complement component C3. Furthermore, *Bpp_ov_* strains were highly susceptible to killing by murine complement, but not by sheep complement. These data demonstrate that the failure of *Bpp_ov_* to colonize mice is due to sensitivity to murine, but not sheep, complement, providing a mechanistic example of how specialization that accompanies expansion in one host can limit host range.

## Introduction

From bacteria to nematodes, pathogens and parasites can vary in host range from very broad (cross-kingdom) to highly specific (single subspecies). Although understanding the mechanistic basis for host specificity is a central biological question and is critical for managing the continual emergence of zoonoses, it remains undefined for most pathogens. Adherence to specific hosts, tissues or cells via receptor-ligand interactions is a well-demonstrated mechanism [1,2,3,4,5]. For example, human pathogen *Listeria monocytogenes* enters host cells via the binding of the bacterial protein internalin to human E-cadherin, but does not infect mice due to a single amino acid difference in mouse E-cadherin [3,4]. The simplicity of receptor-ligand mediated adherence specificity as an explanation for host limitations has led others to propose similar mechanisms to explain host specificity of various pathogens, such as *Bordetella* species [6]. However, there are many steps necessary for a pathogen to successfully colonize, grow, cause disease, and spread, any of which could be the basis for its observed host specificity.

The classical bordetellae are closely related species of respiratory pathogens that differ in host range [6,7,8]. *Bordetella bronchiseptica* infects the widest range of mammalian hosts, causing disease ranging from asymptomatic infection to lethal pneumonia, while *Bordetella pertussis* and *Bordetella parapertussis* are host-restricted pathogens [9,10]. *B*. *pertussis* is limited to humans, causing whooping cough [9,10]. The species *B*. *parapertussis* is comprised of two genetically distinct lineages: *B*. *parapertussis*_*hu*_ (*Bpp*_*hu*_), which is only known to naturally infect humans, and *B*. *parapertussis*_*ov*_ (*Bpp*_*ov*_), which has been recovered only from sheep [11]. While *Bpp*_*hu*_ causes whooping cough in humans, *Bpp*_*ov*_ has been isolated from the lungs of sheep displaying chronic non-progressive pneumonia, as well as from the lungs of healthy sheep [11,12]. Infection with *Bpp*_*ov*_ has also been shown to facilitate the colonization of other known sheep pathogens, such as *Pasteurella haemolytica* [13], which can result in culling of diseased sheep and agricultural losses.

The genome of a *Bpp*_*ov*_ isolate Bpp5 was recently sequenced revealing a remarkably high level of sequence similarity and limited evidence of acquisition of new genes in comparison to the other classical bordetellae, leaving genome reduction as the likely explanation for the phenotypic differences observed between lineages [6,14]. Transcriptomics and comparative genomics analyses have been used to relate gene presence/absence and expression of sets of genes to specific phenotypes. Although some lineages are only observed to naturally infect a single host and therefore are believed to have restricted host ranges, such as *B*. *pertussis* to humans, they still retain the ability to efficiently colonize mice, allowing for direct comparisons in a common *in vivo* experimental system [11]. In the mouse model, identified differences in virulence characteristics can be attributed to specific genes or their differential expression in particular lineages, providing a link between phenotypes and candidate genes involved [15,16]. Intriguingly, every classical bordetellae strain previously described can colonize and grow within the respiratory tracts of mice, including human isolates of the subspecies *Bpp*_*hu*_, with a single exception. Only ovine isolates of *Bpp*_*ov*_ fail to colonize and grow in mice, revealing an example of a limitation of host specificity in an experimental system in which a combination of comparative genomics and mouse molecular immunology can be employed to examine its molecular basis.

Here we examine the basis for the differential ability to successfully colonize the mouse respiratory tract by different lineages of classical bordetellae. Broadly disparate lineages were able to efficiently colonize and grow in mice, including *Bpp*_*hu*_, but *Bpp*_*ov*_ strains were defective. Although these two lineages share most known adhesins and virulence factors, we observed differences in complement resistance factors in the bordetellae, and specifically in *Bpp*_ov_, difference in the locus encoding for the enzymes involved in assembly of the O-antigen component of LPS. *Bpp*_*ov*_ strains lack a detectable O-antigen and were highly susceptible to *in vitro* killing by mouse complement. *Bpp*_*ov*_ efficiently colonized the respiratory tracts of mice lacking complement component 3 (C3), indicating that complement contributes to the rapid clearance of this pathogen. As *Bpp*_*ov*_ strains were not susceptible to sheep complement and efficiently colonize and cause disease in sheep, the loss of O-antigen does not appear to negatively impact its success in its primary host, but the loss of resistance to other host complement is sufficient to explain the observed limitation of this lineage to sheep. These data provide an example of adaptation to host-specific innate immune functions that can result in limitation of host range.

## Materials and Methods

### Ethics Statement

This study was carried out in accordance with the recommendations in the Guide for the Care and Use of Laboratory Animals of the National Institutes of Health. The Institutional Animal Care and Use Committee (IACUC) at The Pennsylvania State University, University Park, PA, approved all protocols (#31297 Bordetella-Host Interactions). Isoflurane was used to anesthetize all animals, and carbon dioxide inhalation was used to euthanize animals in order to minimize suffering.

### Bacterial Strains and growth

*B*. *bronchiseptica* strain RB50 is a rabbit isolate, RB50Δ*wbmBCD* is a derivative of RB50 lacking O-antigen, and *B*. *parapertussis*_*hu*_ strain 12822 was isolated from German clinical trials previously described [17,18]. *B*. *parapertussis*_*ov*_ strains Bpp5 and HI were isolated from sheep in New Zealand and Scotland, respectively, and have been previously described [14]. Bacteria were maintained on Bordet-Gengou (BG) agar (Difco, Franklin Lakes, NJ) containing 10% sheep blood (Hema Resources, Aurora OR) and 20 μg/mL streptomycin (Sigma Aldrich, St. Louis, MO). Liquid cultures were grown at 37°C overnight in a shaker to mid-log phase in Stainer-Scholte (SS) broth with heptakis [16,19].

### Genome-wide SNP tree

Full genome assemblies of *B*. *bronchiseptica* 253 (GenBank ID: HE965806), *B*. *bronchiseptica* MO149 (HE965807.1), *B*. *bronchiseptica* 1289 (GenBank ID: HE983626), *B*. *parapertussis* Bpp5 (GenBank ID: HE965803.1), *B*. *parapertussis* 12822 (GenBank ID: BX470249.1), and *B*. *pertussis* (GenBank ID: BX470248.1) were each processed into 54-bp-long DNA reads and separately mapped against *Bordetella bronchiseptica* RB50 reference genome (GenBank ID: BX470250.1), using SSAHA2 [18]. Then, the alignment served as an input file for locally installed RAxML v7.0.4 [20]to produce a maximum likelihood tree. The raxmlHPC performed rapid bootstrapping (100 bootstrap replicates) using the GTRCAT model for nucleotide substitution, followed by Maximum Likelihood (ML) search using the general time-reversible model for nucleotide substitution with Gamma-distributed rate heterogeneity, or GTRGAMMA. The ML tree was visualized with FigTree v.1.4 (http://tree.bio.ed.ac.uk/software/figtree/).

### Animal Experiments

C57BL/6 mice were obtained from Jackson Laboratories (Bar Harbor, ME). C3 knockout (C3^–/–^) mice were a kind gift from Rick Wetsel and have previously been described [15]. Mice were bred in *Bordetella*-free, specific pathogen-free breeding rooms at The Pennsylvania State University. All animal experiments were performed in accordance to institutional guidelines. Briefly, 4 to 6 week-old mice were lightly sedated with 5% isoflurane (IsoFlo, Abbott Laboratories) in oxygen. 5 x 10^5^ CFU in 50 μl of phosphate-buffered saline (PBS) (Omnipur, Gibbstown, NJ) were pipetted onto the external nares. This method reliably distributes the bacteria throughout the respiratory tract [21]. To quantify bacterial numbers, mice were sacrificed on the indicated time points, and the lungs, trachea, and nasal cavities were excised. Organs were homogenized in PBS, the appropriate dilution was plated on BG agar, and CFU numbers were determined by counting colonies. For collection of vaccine-induced serum, animals were vaccinated intraperitoneal with 10^4^ CFU of heat-killed *Bpp*_*ov*_ strain Bpp5 (incubation for 30 minutes at 65°C) on days 0 and 14, and then bled orbitally on day 28 post-vaccination as previously described [22]. To obtain serum, blood was incubated at room temperature for 30 minutes and centrifuged for 5 minutes at 250 x *g*.

### Comparative protein sequence analysis

Gene products of the O-antigen locus of *B*. *bronchiseptica* strain RB50 and *B*. *parapertussis*_*hu*_ strain 12822 were obtained from the National Center for Biotechnology Information, or NCBI (http://www.ncbi.nlm.nih.gov), while those in *B*. *parapertussis*_*ov*_ strain Bpp5 were obtained from recently sequenced and annotated Bpp5 genome at Sanger and the Pennsylvania State University [9,19]. The amino acid sequence similarity was determined by comparing *B*. *parapertussis*_*hu*_ strain 12822 genes to orthologous genes in RB50 and Bpp5 using the online NCBI protein BLAST search (http://www.ncbi.nlm.nih.gov/BLAST).

### LPS Purification

LPS was purified by a modified Westphal method [23]. 500 mL cultures were seeded with mid-log phase bordetellae and grown in a shaking incubator at 37°C. Cultures were grown in Stainer-Scholte broth with heptakis to an OD_600_ of 0.75. Cells were then pelleted at 500 x *g* and resuspended in 10 mL of endotoxin-free water. An equal volume of 90% w/v phenol was added and the samples were heated to 65°C for 1 hour with stirring. Samples were pre-chilled and centrifuged at 1,000 x *g* and the aqueous phase dialyzed against ddH_2_O for 48 hours. The samples were lyophilized, and the resulting material was resuspended in Tris buffer (pH 7.5) and treated with RNase (Ambion, Austin, TX) and DNase (Mo Bio, Carlsbad, CA) to concentrations of 25 and 100 μg/mL, respectively. Proteinase K (Ambion, Austin, TX) was then added to 100 μg/mL. Following phenol extraction, the aqueous phase was dialyzed for 12 hours against ddH_2_O and lyophilized. Resulting LPS was suspended in endotoxin-free water. Purified LPS was resuspended to a final concentration of 250 μg/mL in Laemmli sample buffer, and separated by SDS-PAGE on a Mini-PROTEAN TGX 4–20% gradient pre-cast gel (Bio-Rad, Hercules, CA). The gel was run and stained using the Pro-Q Emerald 300 Lipopolysaccharide Gel Stain Kit (Invitrogen, Carlsbad, CA) according to the manufacturer’s instructions and visualized with a ChemiDoc XRS Trans UV gel camera.

### Western Immunoblots

Lysates were prepared by diluting 1 mg/mL of purified LPS in 100 μL of Laemmli sample buffer. 10 μg of LPS samples were run on a 10% sodium dodecyl sulfate-polyacrylamide electrophoresis gels in denaturing conditions and transferred to a polyvinylidene difluoride membrane (Millipore, Bedford, MA). Membranes were probed with serum from *B*. *bronchiseptica* (RB50 or 1289) inoculated, or *Bpp*_*ov*_-heat-killed vaccinated mice at the following dilutions: 1:1,000, 1:500, and 1:1,000, respectively. A 1:10,000 dilution of goat anti-mouse Ig HRP conjugated antibody (Southern Biotech, Birmingham, AL) was used as the detector antibody. Membranes were visualized with ECL Western blotting detection reagents (Amersham Biosciences, Piscataway, NJ).

### Serum Killing Assays

Complement killing assays were performed as previously described [18]. Briefly, blood collected from C57BL/6 mice or C3^-/-^ mice, was pooled, incubated at room temperature for 15 minutes and centrifuged at 250 x *g* for 10 min. Sheep serum was obtained from Innovative Research (Novi, MI). Complement-depleted sheep serum was obtained by treating with 0.5 mg/mL of cobra venom factor (CVF) for 30 minutes at 37°C. Approximately 1,000 CFU of RB50, Bpp5, HI, 12822, and RB50Δ*wbm* from mid-log-phase cultures were incubated with the indicated concentration of serum or PBS, or PBS containing CVF for 1 hour at 37°C. Bacterial numbers before and after incubation were determined by plating and CFU counts.

### Complement Deposition

Approximately 10^8^ CFU were taken from a mid-log phase culture and incubated in the absence or presence of 20% complement sufficient or deficient mouse serum for 30 minutes. After two washes with cold PBS, bacteria were resuspended in the absence or presence of FITC conjugated anti-mouse C3b antibodies (1:1000) (eBioscience, San Diego, CA) for 15 minutes on ice in the dark. Bacteria were washed twice with cold PBS and resuspended in 4% paraformaldehyde until acquisition with a Becton Dickinson FC500. Data analysis was performed using FlowJo 7.6.1 software.

### Growth Curve

Overnight cultures grown to mid-log phase were normalized and inoculated into Stainer-Scholte broth with heptakis at an approximate CFU of 10^7^/mL. Cultures were then grown at 37°C with shaking for the duration of the experiments. At the indicated times, samples were removed from the cultures, the OD_600_ read to approximate culture growth, and then plated onto BG agar plates in order to enumerate viable colonies.

### Statistics

For all appropriate data, the average +/- the standard error (error bars) was determined. Results were analyzed using analysis of variance with Tukey simultaneous test for significance or a general linear model in Minitab v. 16 (State College, PA). A *p* value of ≤0.05 was considered significant.

## Results

### *B*. *parapertussis*_ov_ strains are rapidly cleared from the mouse respiratory tract

**Fig 1:**
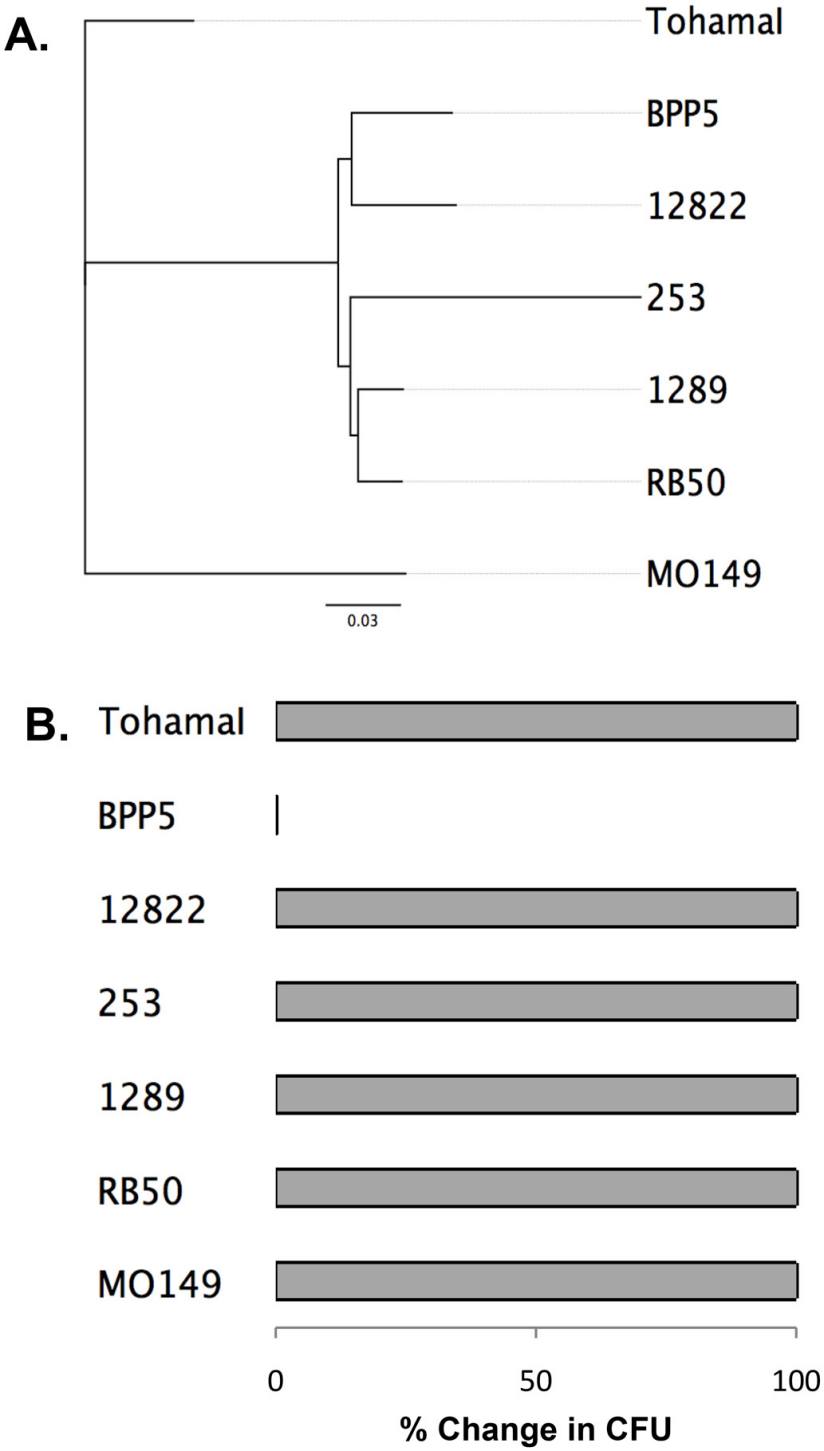
Maximum likelihood genome-wide SNP tree and murine lung colonization of the classical bordetellae. (A) Genomes of bordetellae *B*. *pertussis* strains Tohama I, *B*. *parapertussis* strains Bpp5, 12822, and *B*. *bronchiseptica* strains 253, 1289, MO149 were mapped against reference genome RB50. The ML tree (100 bootstrap replicates, 50% bootstrap cut-off) was estimated with RAxML. (B) Bacterial numbers are represented as percent change in the average CFU from day 0 to 7 post-inoculation (100% recovery cut-off) of three to four mice per time point.

The classical *Bordetella* species have been isolated from a variety of mammalian hosts, and the majority of which have been shown to efficiently infect mice (Fig 1B), [21]. Using a genome-wide SNP tree to determine relatedness of the classical bordetellae (Fig 1A), we compared their ability to efficiently colonize and persist within the lungs of mice through the first week of infection (percent change in CFU from day 0 to day 7 post-inoculation). Consistent with previous findings, strains from both *B*. *bronchiseptica* and *B*. *pertussis* lineages efficiently colonized mice, and at least 100% of CFUs delivered on day 0 were recoverable 7 days post-inoculation [21]. In contrast although *Bpp*_*ov*_ and *Bpp*_*hu*_ are closely related, their ability to colonize murine lungs is vastly different (Fig 1B). Less than 1% of the numbers of *Bpp*_*ov*_ strain Bpp5 delivered on day 0 were recovered from the lungs on day 7, indicating that Bpp5 poorly infects mice in comparison to other classical bordetellae. To confirm that this phenotype is not unique to this strain, we also examined another *Bpp*_*ov*_ strain, HI, which was similarly cleared from the mouse lower respiratory tract within 7 days, suggesting that the failure to colonize mice is a phenotype common to *Bpp*_*ov*_ strains (S1 Fig). The rapid clearance of these *Bpp*_*ov*_ strains suggests they lack one or more factors that are required for efficient colonization of mice, but presumably not sheep.

### Genomic analysis of virulence factor genes of *Bpp*_ov_ strains

**Fig 2:**
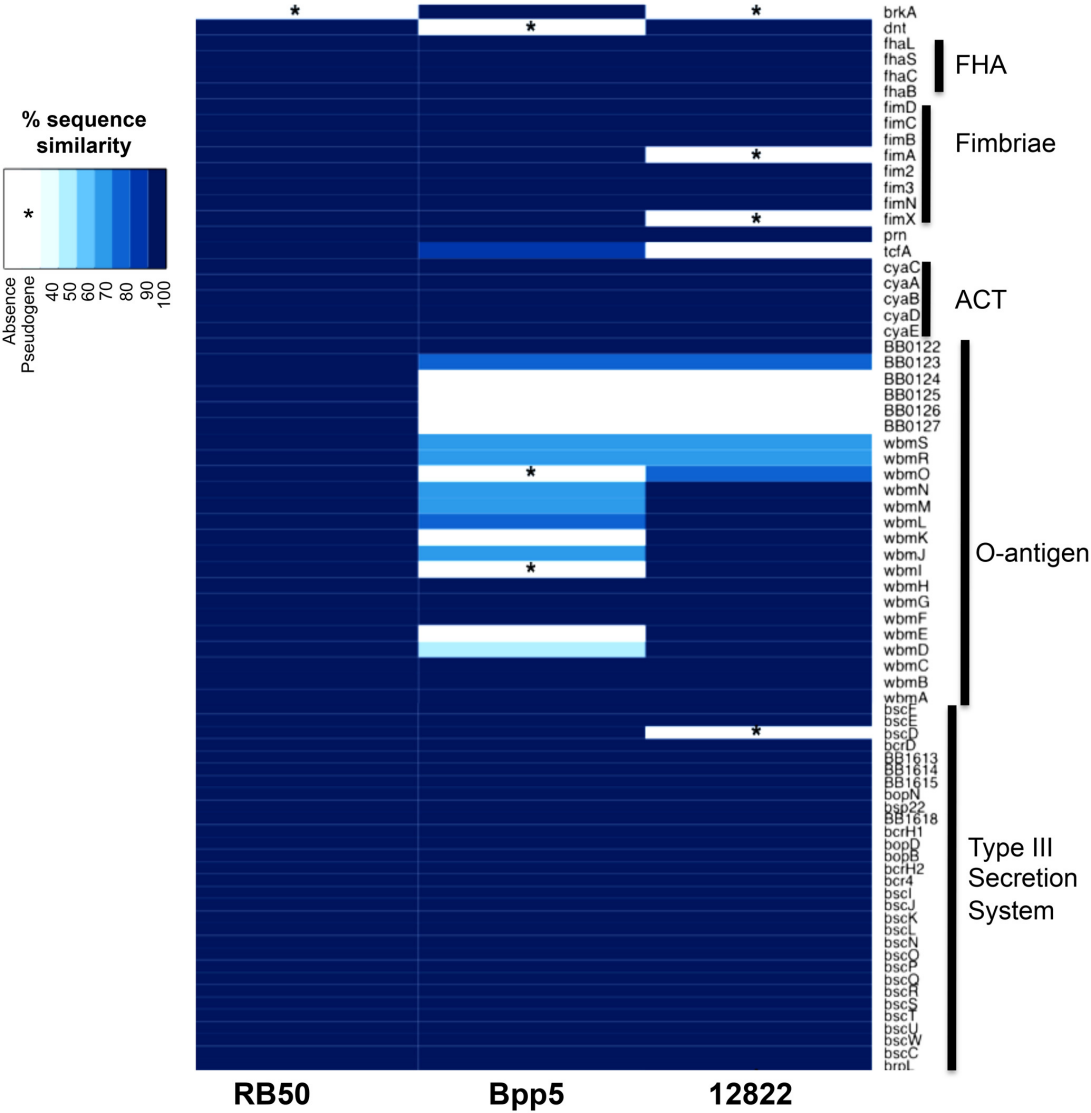
Sequence similarity of *Bordetella* Virulence Factors. Percent sequence similarity of virulence factor genes of *B*. *pertussis* strains (Tohama I, 18323, CS), *B*. *bronchiseptica* strains (253, 1289, MO149, R77, D445) *B*. *parapertussis*_*hu*_ strain 12822 and *B*. *parapertussis*_*ov*_ strain Bpp5 compared to *B*. *bronchiseptica* strain RB50.

*B*. *pertussis* and *Bpp*_*hu*_ have evolved largely through genome loss, which is thought to contribute to the adaptation of both subspecies to humans [9,19]. Recent genome analysis revealed 96% sequence similarity of *Bpp*_*ov*_ strain Bpp5 with the other classical bordetellae genomes, but also indicated a high level of gene inactivation (389 pseudogenes), suggesting that genome reduction likely plays a part in its adaptation to sheep and may contribute to its failure to colonize mice [9]. We therefore used comparative genomic analysis to compare virulence factor genes in *B*. *bronchiseptica* and *B*. *parapertussis* strains that correlate with the ability to colonize mice. Since defects in adherence can lead to rapid clearance, we examined the relative conservation of genes known to be involved in adherence [6,22,24]. Bpp5 has intact genes encoding known adherence factors, such as filamentous hemaglutinin (*fhaB*, *fhaL*, *fhaC*, *fhaS*), pertactin (*prn*) and fimbriae (*fimD*, *fimC*, *fimB*, *fimA*, *fim2*, *fim3*, *fimN*, *fimX*) [11,17,25], and the sequence similarity was greater than 90% in comparison to *B*. *bronchiseptica* strain RB50, suggesting that lack of adherence factors does not explain rapid clearance from mice (Fig 2). Evading the innate immune response is also critical for initial colonization by pathogens. Virulence factors, such as the Type III Secretion System (TTSS) and Adenylate Cyclase Toxin-hemolysin (ACT), have been shown to be important for overcoming aspects of the innate immune response and crucial for efficient colonization over the first seven days of infection [6,26]. Bpp5 has intact and likely functional copies of all the genes for ACT and the TTSS [6] (Fig 2). In comparing other virulence factors genes, we found that several O-antigen genes were missing in the *Bpp*_*ov*_ strain Bpp5 locus compared to *B*. *bronchiseptica* strain RB50 and *Bpp*_*hu*_ strain 12822 (Fig 2) O-antigen loci, suggesting the O-antigen locus may play a role in the rapid clearance of *Bpp*_*ov*_ strains from the mouse lower respiratory tract.

### Bpp5 does not produce an O-antigen

**Fig 3:**
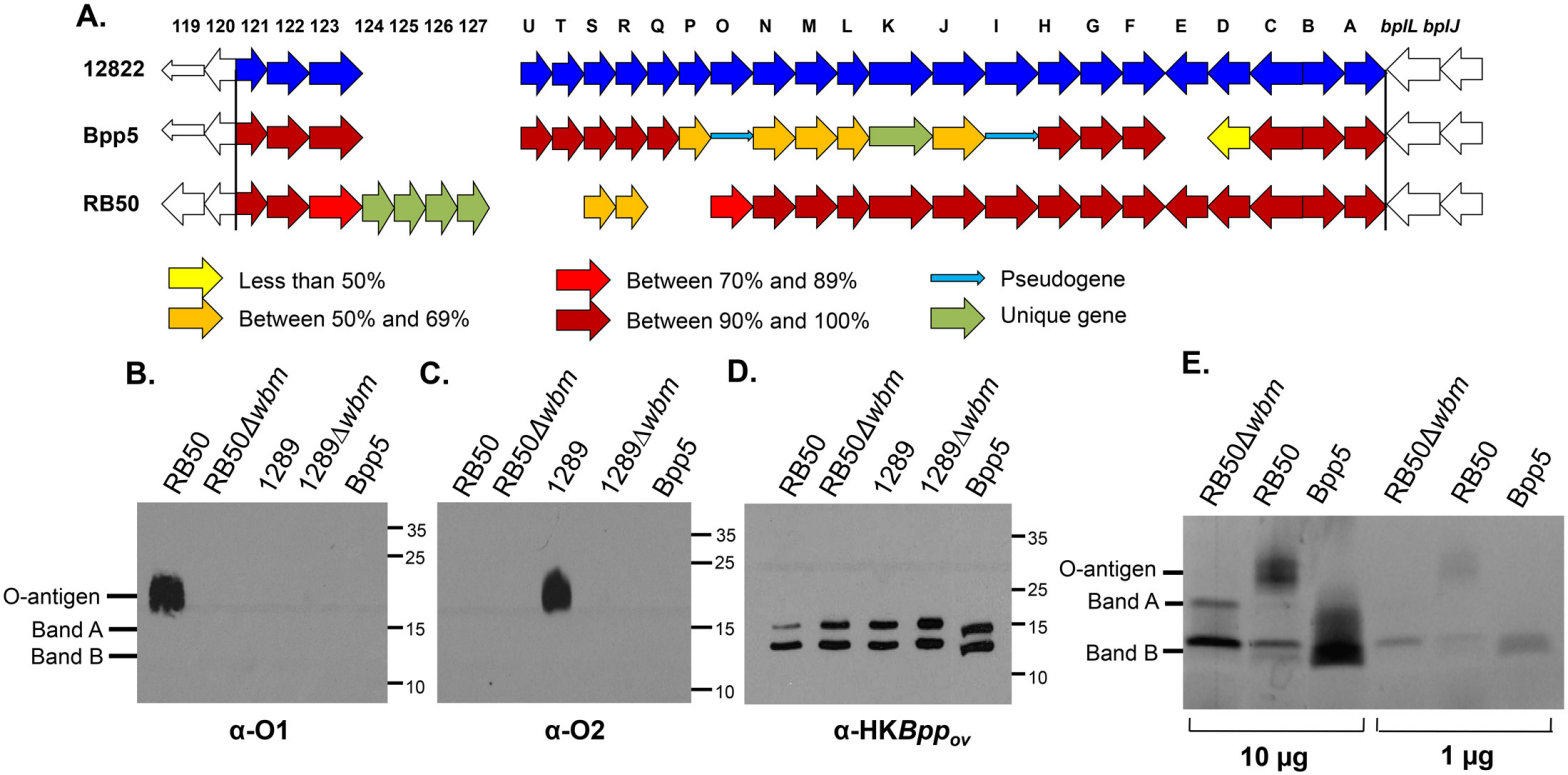
Bpp5 O-antigen locus sequence similarity and production. **(A)** Percent sequence similarity of genes within the O-antigen locus of *B*. *parapertussis*_*hu*_ strain 12822 and *B*. *parapertussis*_*ov*_ strain Bpp5 compared to *B*. *bronchiseptica* strain RB50. Western blots of *B*. *bronchiseptica* strains RB50, RB50Δ*wbm*, 1289, 1289Δ*wbm*, or *B*. *parapertussis*_*ov*_ strain Bpp5 purified LPS probed with convalescent serum from mice inoculated with 5x10^5^ CFU of either RB50 (B) or 1289 (C), or vaccinated with 1x10^8^ CFU of heat-killed Bpp5 (D). (E) Emerald Green stain of purified LPS of *B*. *bronchiseptica* strain RB50, mutant RB50Δ*wbm*, or *B*. *parapertussis*_*ov*_ strain Bpp5.

Since the O-antigen locus has previously been shown to be important for colonization of both *B*. *bronchiseptica* and *Bpp*_*hu*_ strains, we further analyzed divergent genes between *Bpp*_*ov*_ strain Bpp5, and *B*. *bronchiseptica* strain RB50 and *Bpp*_*hu*_ strain 12822. The O-antigen loci in both *B*. *bronchiseptica* and *Bpp*_*hu*_ contain 24 genes (BB0121 to BB0144/ BPP0121 to BPP0144)[6,27], but genes predicted to encode modifications to the polysaccharide backbone (*wbmP*, *wbmN*, *wbmM*, *wbmL*, *wbmJ*, and *wbmD*) are less conserved in *Bpp*_*ov*_ strain Bpp5 in comparison to those genes in *Bpp*_*hu*_ strain 12822 and *B*. *bronchiseptica* strain RB50 (Fig 3A), consistent with previous CGH analysis [28]. Also, *wbmO* and *wbmI* are predicted to be pseudogenes due to frame-shift mutations, consistent with a prior prediction for *wbmI* ([17], Fig 3A). Additionally, *wbmE* is completely missing in Bpp5 (Fig 3A) and *wbmK* is replaced by a unique gene, with closest sequence similarity to a gene that encodes a methyltransferase type 11 in other bacteria, such as *Wolinella succinogenes* ([28], Fig 3A). Overall, this comparative analysis of the Bpp5 O-antigen locus suggests that Bpp5 has a novel or defective O-antigen.

To examine whether Bpp5 produces an O-antigen similar to previously defined bordetellae O-antigen types [18,24], Bpp5 was probed with antibodies against either *B*. *bronchiseptica* strain RB50 (O1 serotype) or *B*. *bronchiseptica* strain 1289 (O2 serotype). Serum obtained from mice convalescent from *B*. *bronchiseptica* strain RB50 infection recognized O-antigen from RB50, but not any of the other strains or the O-antigen mutants. Notably, RB50 O-antigen antibodies did not cross-react with a Bpp5 O-antigen, indicating that Bpp5 does not share the same (O1-type) O-antigen (Fig 3B). Antibodies raised against *B*. *bronchiseptica* 1289 O-antigen (O2-type) also did not recognize an O-antigen in Bpp5 (Fig 3C), indicating that the *Bpp*_*ov*_ strain Bpp5 does not produce an O-antigen or that it produces one that is antigenically distinct from O1- and O2-type serotypes. Furthermore, *Bpp*_*ov*_ strains probed with serum antibodies from mice vaccinated with heat-killed Bpp5 cross reacted with Band A (the inner core trisaccharide) and Band B (the outer core branched-chain oligosaccharide attaching Lipid A to the O-antigen) forms of LPS, but not a larger form (Fig 3D), suggesting that Bpp5 does not produce an O-antigen molecule or that it produces a molecule that is not immunogenic. To distinguish between these two possibilities, LPS was purified from *B*. *bronchiseptica* strain RB50, RB50Δ*wbm*, or *Bpp*_*ov*_ strain Bpp5 and stained to determine presence or absence of O-antigen, as well as Band A and Band B. At a concentration of either 10μg or 1μg, *B*. *bronchiseptica* strain RB50 LPS produced a readily visible form containing O-antigen, while LPS from RB50Δ*wbm* at either concentration only contained LPS Band A and Band B (Fig 3E). Notably, LPS purified from *Bpp*_*ov*_ strain Bpp5 produced bands that correlated with Band A and B, but there was no form containing O-antigen detected at either LPS concentration (Fig 3E). Together, these data indicate that Bpp5 has a degraded O-antigen locus and does not produce an O-antigen.

### Complement deposition and killing of *Bpp*_*ov*_ strains

**Fig 4:**
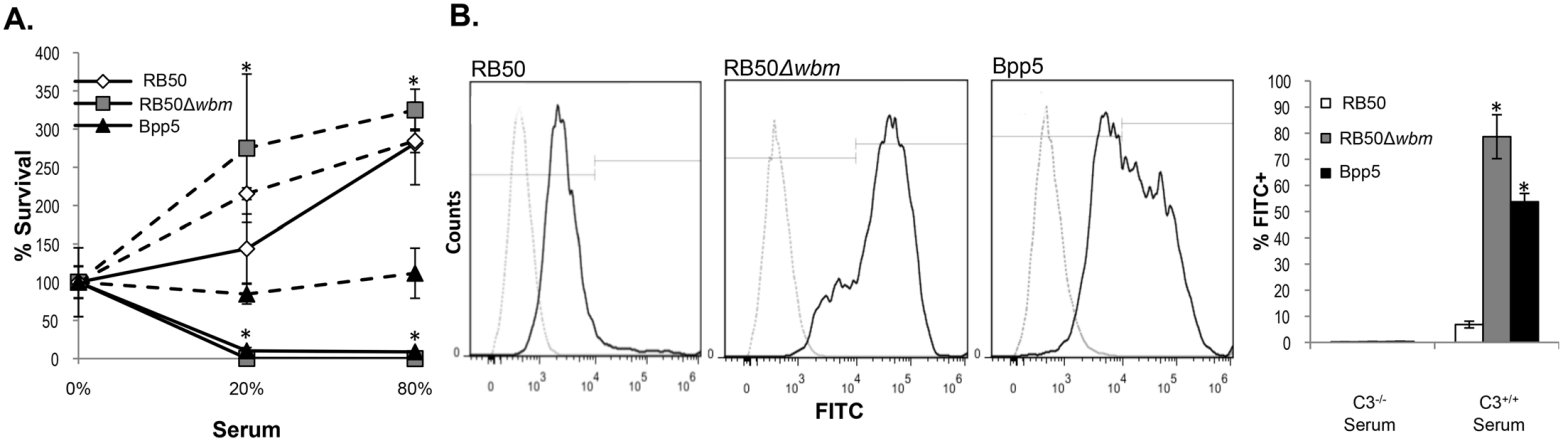
Complement efficiently deposits onto the cell surface and kills Bpp5. (A) *B*. *bronchiseptica* RB50 (diamond), RB50Δ*wbm* (square) or *B*. *parapertussis*_*ov*_ Bpp5 (triangle) were incubated with complement active (solid lines) or complement inactive serum (dashed lines) at the indicated concentrations for 1 hour. The average percent survival of three independent experiments is shown +/- standard error. (B) Flow cytometry analysis of C3b deposition onto RB50, RB50Δ*wbm* or Bpp5 incubated with complement sufficient (solid line) or deficient (dashed line) serum. Samples were unstained or stained with FITC-anti-mouse C3 antibodies and analyzed and a representative image was shown. The average percentage of FITC-positive cells of three replicates is indicated +/- standard error. * indicates *p* value <0.05 in comparison to RB50.

*B*. *bronchiseptica* and *Bpp*_hu_ strains are protected from complement-mediated killing by O-antigen, which blocks complement from depositing onto the bacterial cell surface [18,24]. Since *Bpp*_*ov*_ strains do not appear to have an O-antigen, we determined if complement efficiently deposits onto and kills *Bpp*_*ov*_ strains. In serum killing assays, 100% of RB50 survived in a concentration of 80% mouse serum for 1 hour at 37°C, while serum concentrations as low as 20% efficiently killed RB50Δ*wbm*, demonstrating the previously observed dependence on O-antigen to resist complement [15] (Fig 4A). In comparison, 100% of Bpp5 was killed in 80% serum and less than 20% of Bpp5 survived in 20% serum (Fig 4A). To determine if complement was responsible for the killing, bacteria were incubated in complement-deficient serum (dashed lines), and all three strains survived indicating that killing was mediated by complement.

O-antigen could protect bacteria against complement-mediated killing either by preventing deposition or subsequent complement membrane attack complex formation [18]. To determine if greater killing was due to more complement deposition, bacteria were incubated in mouse complement sufficient and deficient serum, and then analyzed via flow cytometry for complement protein 3(C3b) deposition (Fig 4B). In 20% complement sufficient mouse serum, approximately 7% of *B*. *bronchiseptica* strain RB50 stained FITC-positive for C3b (Fig 4B), while over 80% of RB50Δ*wbm* stained positive, indicating that O-antigen blocks C3b deposition onto the bacterial surface (Fig 4B). 56% of *Bpp*_*ov*_ strain Bpp5 stained FITC-positive for C3b (Fig 4B), which was significantly more C3b-positive bacteria than *B*. *bronchiseptica* strain RB50. These data indicate that Bpp5 does not prevent complement deposition onto the bacterial cell surface.

### Complement contributes to the efficient control of *Bpp*_ov_ strains in mice

**Fig 5:**
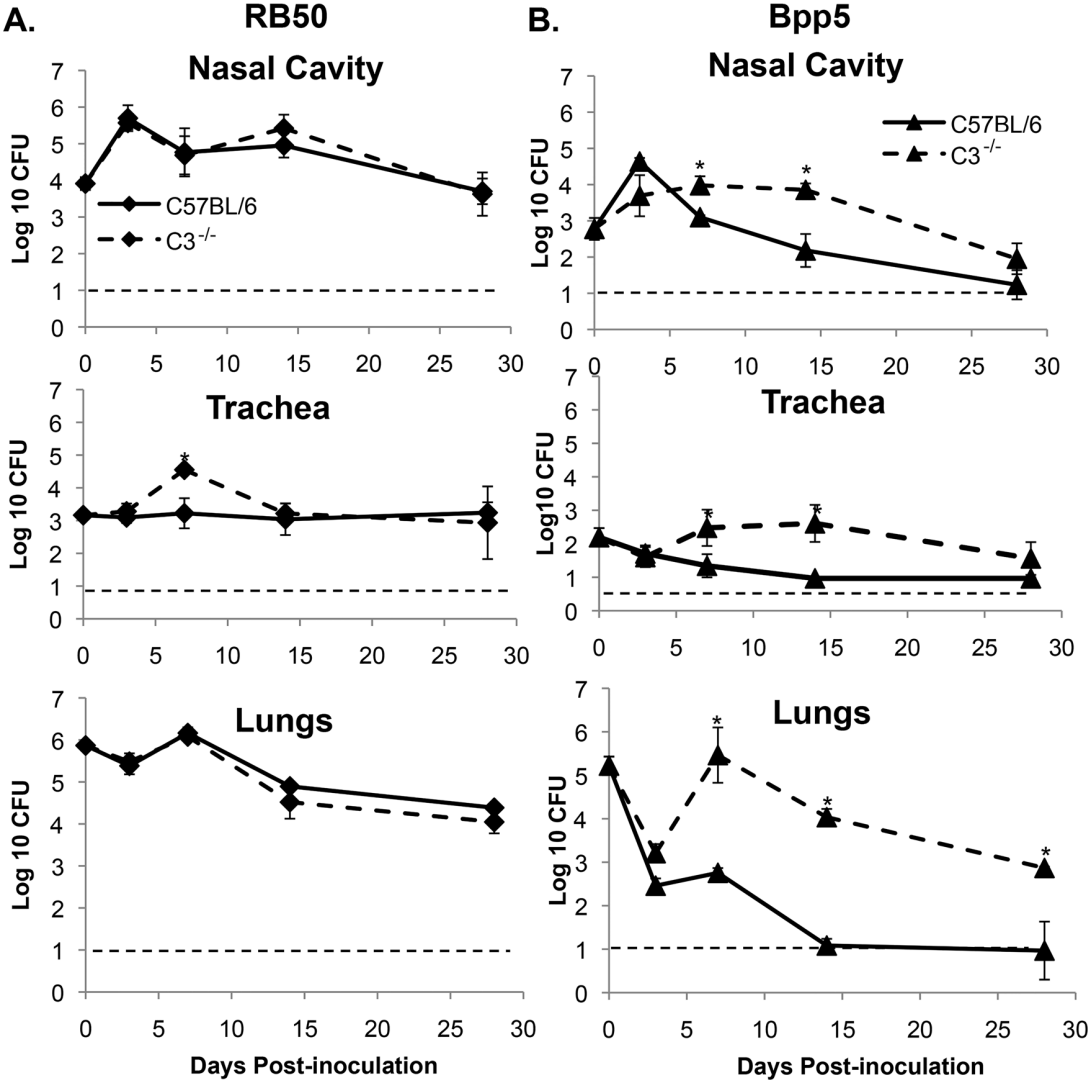
Complement contributes to control of *B*. *parapertussis*_*ov*_ strains. C57BL/6 mice (solid lines) or C3 deficient mice (dashed lines) were inoculated with *B*. *bronchiseptica* strain RB50 (A) or *B*. *parapertussis*_*ov*_ strain Bpp5 (B). Bacterial colonization was enumerated and Log_10_ average of three to four mice per group +/- standard deviation was determined at the indicated time points. Dashed line indicates limit of detection. * indicates *p* value <0.05 between wild-type and C3 deficient groups.

Based on the results above we hypothesized that complement contributes to the efficient control of *Bpp*_ov_ strains in the mouse respiratory tract, leading to the prediction that *Bpp*_*ov*_ should not be defective in mice lacking complement. To test this, wild-type or C3 (complement protein 3) knockout mice were inoculated with 5x10^5^ CFU of *B*. *bronchiseptica* strain RB50 or *Bpp*_*ov*_ strain Bpp5. Numbers of RB50 within the respiratory tracts of wild-type mice or complement deficient mice were not significantly different since O-antigen protects against complement-mediated killing of RB50, as previously observed [24] (Fig 5A). However, *Bpp*_*ov*_ strain Bpp5 colonized the nasal cavity and tracheas of C3 deficient mice much more efficiently than wild-type mice, being recovered at approximately 10-fold higher numbers on both days 7 and 14 post-inoculation (Fig 5B). *Bpp*_*ov*_ strain Bpp5 also efficiently colonized lungs of C3^-/-^ mice on days 7, 14, and 28 post-inoculation at numbers approximately 100-fold to 1000-fold higher than in wild-type mice (Fig 5B). However, the colonization of *Bpp*_*ov*_ strain Bpp5 in C3^-/-^ mice is still attenuated relative to RB50, indicating that complement is not the only mechanism by which mice control *Bpp*_*ov*_ strains. Together, these data indicate that the primary defect of *Bpp*_*ov*_ in the respiratory tracts of mice is due to its sensitivity to complement.

### Sheep serum does not kill Bpp5

**Fig 6:**
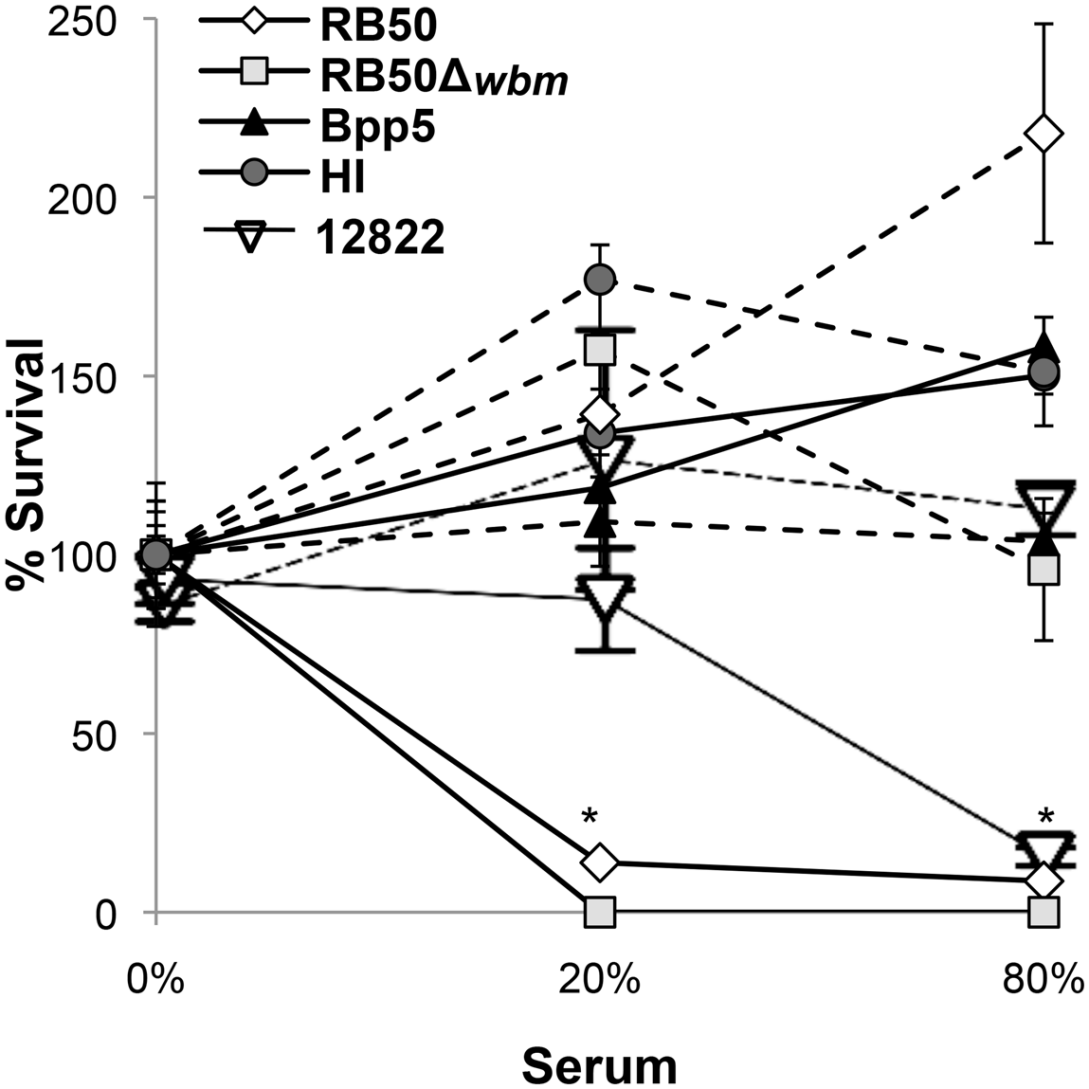
Sheep serum does not kill Bpp5. *B*. *bronchiseptica* RB50 (diamond), RB50Δ*wbm* (square), *B*. *parapertussis*_*hu*_ 12822 (empty triangle) or *B*. *parapertussis*_*ov*_ Bpp5 (filled triangle) or HI (circle) were incubated with PBS (solid lines) or CVF treated (dashed lines) sheep serum for 1 hour at the indicated concentrations. The average percent survival of three independent experiments is shown +/- standard error. * indicates a *p* value of ≤0.05 between *B*. *parapertussis*_*ov*_ strains and *B*. *bronchiseptica* strain RB50.

Complement is an evolutionarily conserved innate immune defense maintained among vertebrates and even invertebrates [1]. *Bpp*_*ov*_ does not efficiently colonize mice due to its sensitivity to mouse complement, yet is a successful sheep pathogen; raising the possibility that differences between mouse and sheep complement could contribute to the host specificity of *Bpp*_*ov*_. We therefore hypothesized that *Bpp*_ov_ would be able to survive in the presence of sheep complement. To compare their resistance to sheep complement, we incubated mid-log phase bacteria in sheep serum (Fig 6). Approximately 90% of RB50 and nearly 100% of RB50Δ*wbm* were killed in 20% or 80% sheep serum, but not serum depleted of complement, indicating that *B*. *bronchiseptica* strain RB50 is sensitive to sheep, but not mouse, complement (Fig 6). Intriguingly, Bpp5 and HI survived in 20% and 80% sheep serum and even had growth in 80% serum (Fig 6), showing that although it is efficiently killed in mouse serum, *Bpp*_*ov*_ strains survive in the serum of sheep, their natural host. In contrast, approximately 90% of the *Bpp*_*hu*_ strain 12822 was killed in the 80% sheep serum, but not in the complement deplete serum or the 20% serum. This suggests that resistance to sheep serum is not conserved in all *B*. *parapertussis* strains, but rather is restricted to *Bpp*_*ov*_ strains. Together these data suggest that in adapting to sheep *Bpp*_*ov*_ has gained the ability to resist sheep complement, but lost the ability to resist the complement of mice, potentially explaining the apparent host-restriction of this clade.

## Discussion

Understanding the mechanistic basis for changes in host specificity is critical to our management of the ongoing threats of zoonoses and newly emerging infectious diseases, most of which crossover from other hosts. Although adaptation of bacterial pathogens to distinct hosts is complex and may be difficult to define, a focus on closely related species with markedly different host ranges present a valuable opportunity to examine the mechanisms involved in host specificity. The classical bordetellae offer a unique experimental system to study host specificity as the three sub-species have differing host ranges, but are closely related based on whole-genome SNP analysis, and genes shared between the sub-species have greater than 95% sequence similarity [9,11,14]. Additionally, since most bordetellae efficiently infect mice, the tools of mouse molecular immunology allow us to probe the contributions of specific immune components to particular aspects of bacterium-host interactions, including those that can limit host range.

Our data indicate that the *Bpp*_*ov*_ strain Bpp5 does not produce an O-antigen molecule (Fig 3E) and does not prevent deposition of mouse complement onto the bacterial surface (Fig 4C), allowing mouse complement-mediated killing. Despite efficient killing of *Bpp*_*ov*_ strains by mouse complement (Fig 4A), sheep complement did not kill either *Bpp*_*ov*_ (Fig 6), reflecting the adaptation of this specific lineage to sheep. The rapid killing of *B*. *bronchiseptica* strain RB50 in sheep serum indicates that sheep are not complement deficient, but rather that their complement differs in some way that *Bpp*_*ov*_ has adapted to, while the *B*. *bronchiseptica* lineage containing RB50 has not. *Bpp*_*ov*_ could have acquired some mechanism to specifically inhibit the complement pathway of sheep that does not inhibit mouse complement. It is also possible that *Bpp*_*ov*_ fails to activate sheep complement, although it lacks an apparent O-antigen, suggesting some alternative mechanism.

Intriguingly, previous work has shown that the classical bordetellae possess several factors that interact with complement, including O-antigen, filamentous hemagglutinin (FHA), and the autotransporters Vag8 and *Bordetella* resistance to killing (BrkA) [20,29,30,31]. However, *Bpp*_*ov*_ strain Bpp5 does not produce an O-antigen, and *vag8* is predicted to be a pseudogene (premature stop codon after ~32% of the gene and a frameshift mutation at C terminus) (Fig 2) [17]. Although FHA has been shown to bind complement-4-binding protein, a negative regulator of complement, there is no evidence that this protects against complement-killing [25]. The only known complement resistance factor *Bpp*_ov_ appears to have intact is *brkA* [6,14]. It is intriguing to note that both *B*. *bronchiseptica* and *Bpp*_*hu*_ strains have the genes encoding O-antigen and Vag8, while *brkA* is a predicted pseudogene, whereas *Bpp*_*ov*_ strain Bpp5 has lost both O-antigen and *vag8*, yet has *brkA*. In adapting to sheep *Bpp*_*ov*_ strains may have lost complement resistance factors that, while they might be important in other hosts, are not required for protection against sheep complement.

The loss of O-antigen within the *Bpp*_*ov*_ clade could provide additional insight into the adaption of other bordetellae sub-species to specific host populations. *B*. *pertussis* has lost the O-antigen locus, and is similarly specialized to a human host. Since the O-antigen is both energetically costly to make and a dominant antigen, the loss of O-antigen may have been beneficial to *B*. *pertussis* [26]. However, *Bpp*_*hu*_ strains that circulate among humans have retained many genes in this large locus [22,32,33], indicating that this locus performs some function for *Bpp*_*hu*_ that is not required by *B*. *pertussis*. Previously, we have shown that loss of altered O-antigen can allow evasion of cross-immunity mediated by antibodies to O-antigen. Potentially contributing to their co-existence in the same ecological niche *Bpp*_*hu*_ has been previously shown to evade *B*. *pertussis*-induced immunity by shielding shared surface antigens behind its O-antigen. While *Bpp*_*ov*_ strains are only found in sheep populations, *B*. *bronchiseptica* strains have also been isolated from sheep [11], and it is therefore possible that the loss of O-antigen production allowed *Bpp*_*ov*_ strains to circulate in a *B*. *bronchiseptica* immune population. This suggests that inter-strain competition may be an important aspect of the evolution of this locus, and raises the interesting possibility that increased success within a single host may result in loss of ability to infect others.

In conclusion, these data reveal an immunological mechanism that explains the observed host specificity of the *Bpp*_*ov*_ lineage and suggests that the differing repertoires of complement resistance factors confer differing susceptibilities to complement of various hosts. As various complement activation pathways are critical aspects of both host immunity to disease and some autoimmune pathologies, understanding the molecular basis for ability of bacteria to either prevent complement activation or inhibit individual pathways is of substantial significance.

## Supporting Information

S1 Fig *B*. *parapertussis*_*ov*_ strains are attenuated in mice. C57BL/6 mice were inoculated with *B*. *parapertussis*_*ov*_ strains Bpp5 and HI. Bacterial colonization was enumerated from the nasal cavity, trachea, and lungs of three to four mice per group at 0, 3, 7, 14, and 28 days post-inoculation. Error bars indicate standard deviation (SD). Dashed line indicates limit of detection.
(TIF)

S2 Fig Growth rate of *B*. *parapertussis* strains. (A) Growth of *B*. *bronchiseptica* strain RB50 (purple), *B*. *parapertussis*_*hu*_ strain 12822 (green) and B. *parapertussis*_*ov*_ strains Bpp5 (blue) and HI (red) in Stainer-Scholte media over time. (B) Doubling time of indicated strains based on growth during mid-log phase. Error bars indicate standard deviation (SD).
(TIF)
