# Supplementary figures and images for: Host Specificity of Ovine Bordetella parapertussis and the Role of Complement
Source: PLoS One. 2015 Jul 9;10(7):e0130964. doi: 10.1371/journal.pone.0130964 (PMC4497623; doi:10.1371/journal.pone.0130964)

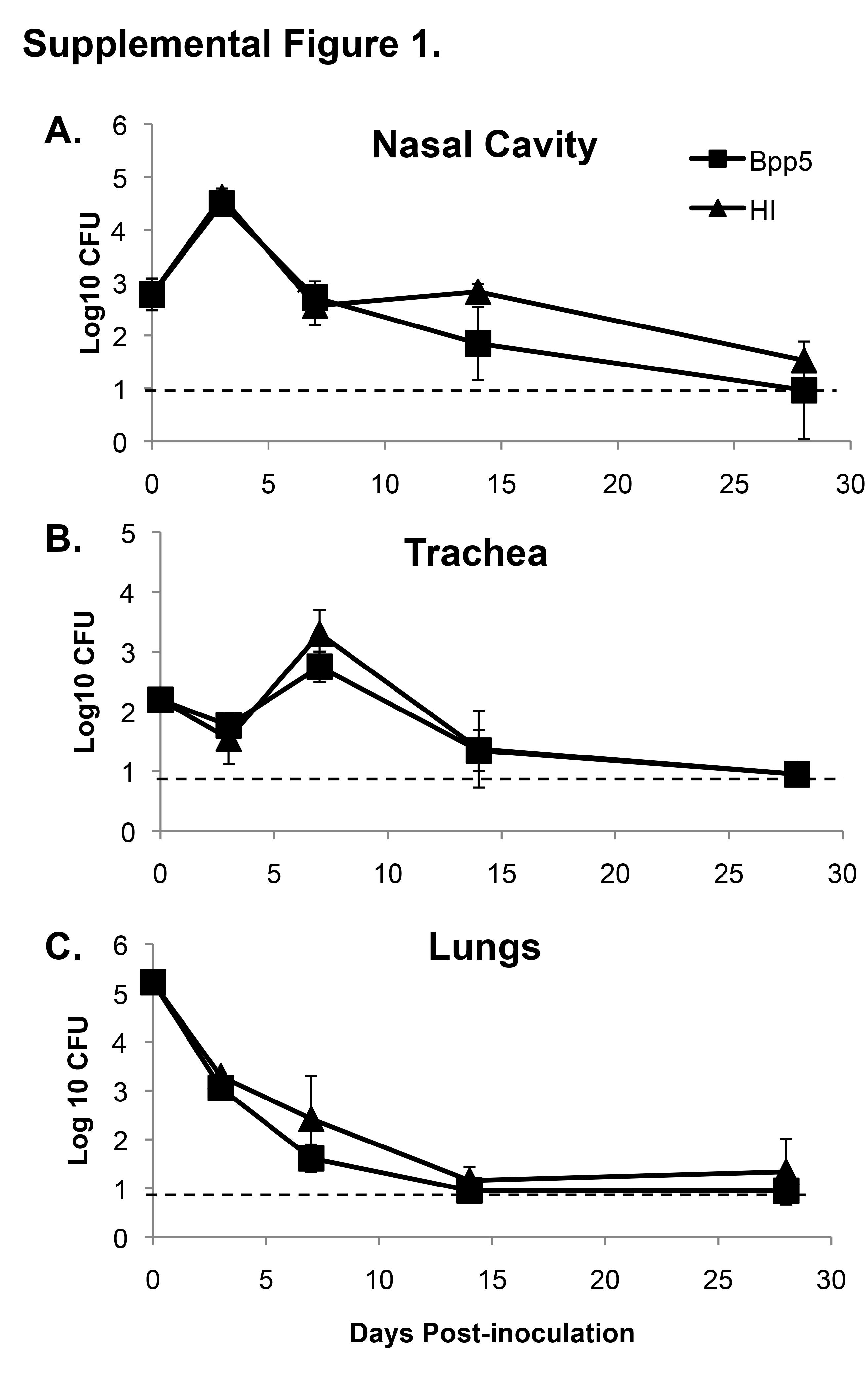

Supplement: S1 Fig — C57BL/6 mice were inoculated with B. parapertussis ov strains Bpp5 and HI. Bacterial colonization was enumerated from the nasal cavity, trachea, and lungs of three to four mice per group at 0, 3, 7, 14, and 28 days post-inoculation. Error bars indicate standard deviation (SD). Dashed line indicates limit of detection. (TIF) [file pone.0130964.s001.tif]

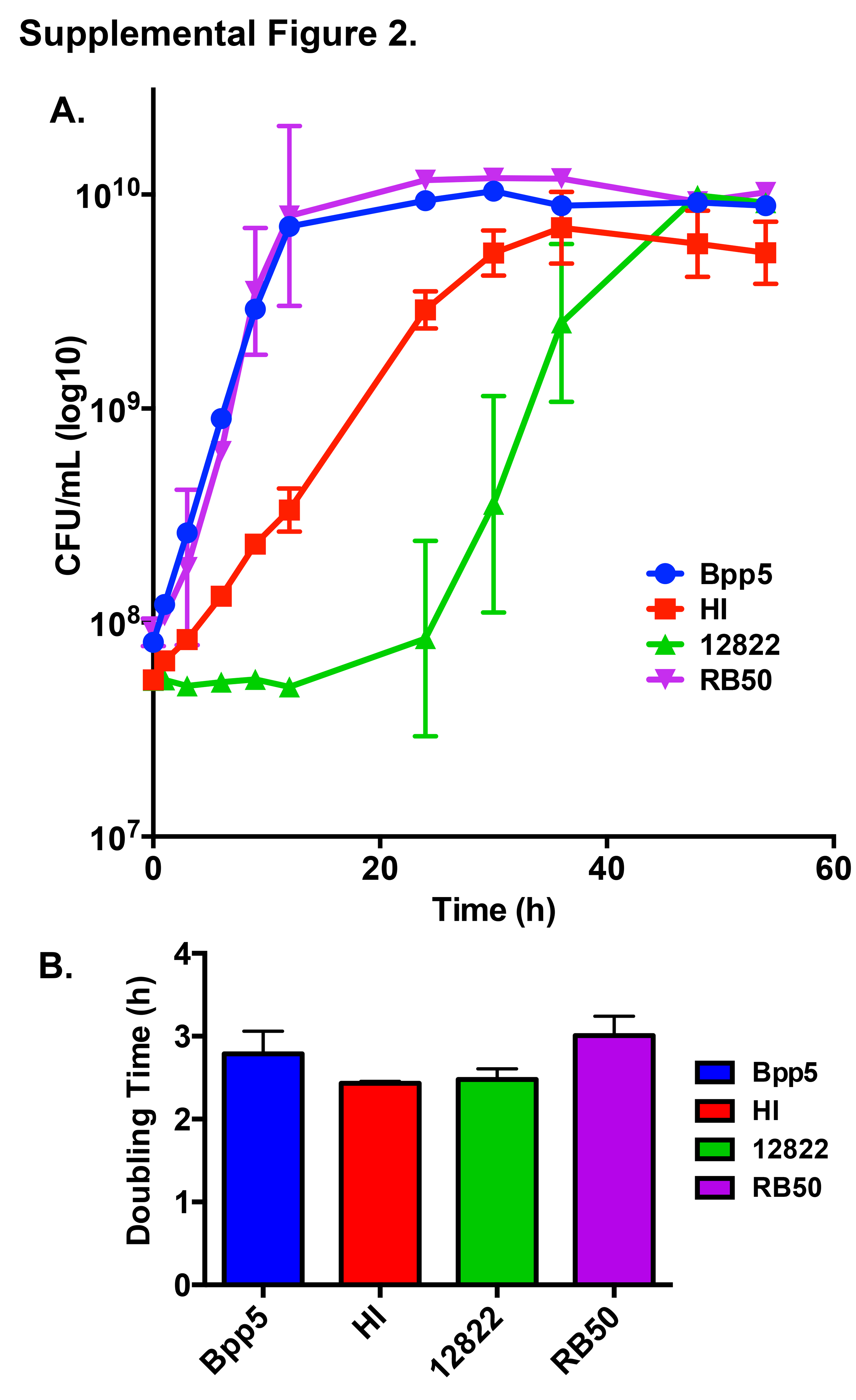

Supplement: S2 Fig — (A) Growth of B. bronchiseptica strain RB50 (purple), B. parapertussis hu strain 12822 (green) and B. parapertussis ov strains Bpp5 (blue) and HI (red) in Stainer-Scholte media over time. (B) Doubling time of indicated strains based on growth during mid-log phase. Error bars indicate standard deviation (SD). (TIF) [file pone.0130964.s002.tif]
